# HMGB1/autophagy pathway mediates the atrophic effect of TGF-β1 in denervated skeletal muscle

**DOI:** 10.1186/s12964-018-0310-6

**Published:** 2018-12-07

**Authors:** Xiaofan Yang, Pingping Xue, Xin Liu, Xiang Xu, Zhenbing Chen

**Affiliations:** 1 Department of Hand Surgery, Union Hospital, Tongji Medical College, Huazhong University of Science and Technology, Wuhan, 430022 China; 2 Department of Pharmacy, Tongji Hospital, Tongji Medical College, Huazhong University of Science and Technology, Wuhan, 430030 China; 3 Department of Anesthesiology, The People’s Hospital of Hanchuan, Renmin Hospital of Wuhan University, Hanchuan, 432300 Hubei Province China

**Keywords:** Denervation, Skeletal muscle, Atrophy, TGF-β1, HMGB1, Autophagy

## Abstract

**Background:**

Transforming growth factor beta 1 (TGF-β1) is a classical modulator of skeletal muscle and regulates several processes, such as myogenesis, regeneration and muscle function in skeletal muscle diseases. Skeletal muscle atrophy, characterized by the loss of muscle strength and mass, is one of the pathological conditions regulated by TGF-β1, but the underlying mechanism involved in the atrophic effects of TGF-β1 is not fully understood.

**Methods:**

Mice sciatic nerve transection model was created and gastrocnemius were analysed by western blot, immunofluorescence staining and fibre diameter quantification after 2 weeks. Exogenous TGF-β1 was administrated and high-mobility group box-1 (HMGB1), autophagy were blocked by siRNA and chloroquine (CQ) respectively to explore the mechanism of the atrophic effect of TGF-β1 in denervated muscle. Similar methods were performed in C2C12 cells.

**Results:**

We found that TGF-β1 was induced in denervated muscle and it could promote atrophy of skeletal muscle both in vivo and in vitro, up-regulated HMGB1 and increased autophagy activity were also detected in denervated muscle and were further promoted by exogenous TGF-β1. The atrophic effect of TGF-β1 could be inhibited when HMGB1/autophagy pathway was blocked.

**Conclusions:**

Thus, our data revealed that TGF-β1 is a vital regulatory factor in denervated skeletal muscle in which HMGB1/ autophagy pathway mediates the atrophic effect of TGF-β1. Our findings confirmed a new pathway in denervation-induced skeletal muscle atrophy and it may be a novel therapeutic target for patients with muscle atrophy after peripheral nerve injury.

**Electronic supplementary material:**

The online version of this article (10.1186/s12964-018-0310-6) contains supplementary material, which is available to authorized users.

## Background

Autophagy is a conserved cellular process by which eukaryotic cells are able to effectively degrade particular cellular components in a regulated manner, thereby recycling vital macromolecules for subsequent metabolic processes [1–3]. This process is facilitated by the localization of autophagic targets such as damaged proteins into organelles known as autophagosomes. These autophagosomes subsequently fuse with lysosomes, leading to the hydrolytic degradation of these targeted proteins [4, 5]. Autophagy also places a key role in regulating mitochondria-associated oxidative stress within cells via mitochondrial degradation. Disruption of autophagic processes within cells can have substantial adverse effects to cellular and tissue function, thereby causing or exacerbating diseases states [6, 7]. LC3B is a key autophagic marker, as it is post-translationally modified from its inactive form (LC3-I) to its active form (LC3-II) – an autophagosome component [8–10]. Another widely used marker for autophagic flux is the autophagy receptor sequestosome 1 (SQSTM1, p62), which physically links autophagic cargo to the autophagic membrane and is itself degraded by autophagy [11–13].

Skeletal muscle atrophy occurs when an individual loses muscle mass in a progressive fashion as a result of a lack of protein homeostasis, owing to decreased protein synthesis and/or increased protein degradation [14, 15]. This pathological atrophic condition manifests itself in many contexts, including aging, cancer, severe malnourishment or denervation [16–20]. Previous studies, primarily in animal models, have demonstrated that skeletal muscle protein degradation is regulated both by autophagy and by the ubiquitin-proteasome system (UPS) [21, 22]. This latter system relies upon the tagging of specific target proteins with ubiquitin molecules, thereby targeting them for proteasomal degradation [23]. While the regulatory networks governing UPS have been extensively characterized [22, 24, 25], those regulating autophagy are still incompletely defined in the context of denervation-induced skeletal muscle atrophy.

TGF-β is a secreted cytokine with complex regulatory activities across a range of tissues and cell types, owing to its ability to regulate the generation of the extracellular matrix (ECM). Originally TGF-β was known to drive fibroblast proliferation and to be capable of driving metastatic cellular transformation [26]. TGF-β1 has also recently been shown to regulate the function and pathology of skeletal muscle, with the potential to induce both fibrosis and skeletal muscle atrophy [27–30]. Although TGF-β1 has been shown to drive a reduction in muscle strength and myotube diameter, the molecular mechanisms underlying these effects are poorly characterized.

In this study, up-regulated TGF-β1 and activation of autophagy were confirmed in denervated skeletal muscles and C2C12 cells. The atrophic effect of TGF-β1 was demonstrated through adding exogenous TGF-β1 and its inhibitor while this effect was attenuated when autophagy was blocked. To further explore the mechanism during skeletal muscle atrophy, RNA sequencing was carried out and HMGB1, which has been demonstrated to be involved in several cellular processes including autophagy [31, 32], was identified to be differentially expressed between normal and atrophic muscles. Then we confirmed the cytosolic and ectocytic translocation of HMGB1 in atrophic muscles and this activity was modulated by TGF-β1. Furthermore, inhibition of HMGB1 by siRNA reversed the activated autophagy and improved atrophy induced by TGF-β1 both in vivo and in vitro. Taken together, we can conclude that TGF-β1 is a vital atrophic factor in denervated skeletal muscle and HMGB1/autophagy pathway actually mediates the atrophic effect of TGF-β1.

## Methods

### Animal procedures

All studies used 8-week old male C57BL/6 J mice housed on a 24 h day-night cycle and purchased from the Animal Experiment Center of Huazhong University of Science and Technology. Denervation was performed surgically as previously described [33]. The mice were randomized into the following groups (5 mice/group): sham operation group (control); denervation group; denervation + 1 mg/kg TGF-β1 (R&D Systems, USA) group; denervation + 1 mg/kg TGF-β1 + 1 mg/kg SB525334 (inhibitor of TGF-β receptor I; MedChemExpress, USA) group; denervation + 1 mg/kg SB525334 group; denervation + 50 mg/kg chloroquine (CQ; chloroquine diphosphate salt, Sigma, USA) group; denervation +siRNA-HMGB1 (RiboBio, China) group. TGF-β1 and SB525334 were dissolved in dimethyl sulfoxide and were injected intraperitoneally daily. CQ was dissolved in physiological saline and were injected intraperitoneally 4 times a week according to Ikezoe et al. [34]. Mice were euthanized at the indicated time, and gastrocnemius and tibialis anterior (TA) were removed, weighed, frozen and fixed to assess atrophy.

Sciatic nerve transection surgeries were conducted on the right hind legs of mice anesthetized using 40 mg/kg 2% sodium pentobarbital injected intraperitoneally. The sciatic nerve was identified and elevated via a lateral incision to the mid-thigh. A resection of a 0.5 cm long portion of the sciatic nerve was then performed, the two ends were buried in muscles, then the incision sites were closed using 4–0 absorbable sutures.

All experimental procedures were in accordance with the guidelines of the Chinese National Institutes of Health. Experimental protocols were approved by the Ethical Committee on Animal Experiments (Huazhong University of Science and Technology).

### Cell culture

Briefly, C2C12 cells (mouse myoblast cell line; iCell Bioscience Inc., China) were grown in high-glucose Dulbecco’s modified Eagle’s medium (DMEM; Gibco, USA) supplemented with 10% fetal bovine serum (FBS; Gibco, USA), 100 U/ml of penicillin, and 100 μg/ml of streptomycin in a 5% CO2 humidified atmosphere at 37 °C. The cells were differentiated into myotubes after incubated in differentiation medium (2% horse serum in DMEM) for 7 days as described previously [35]. To evaluate the atrophic effect of TGF-β1, the myotubes were then incubated with a concentration gradient of TGF-β1 for the next 72 h. To block the TGF-β pathway, cells were pre-incubated for 1 h with the inhibitor SB525334 (5 μM). To prevent acidification of lysosomes and inhibits the final stages of autophagic flux induced by TGF-β1, cells were pre-incubated with CQ (10 μM) 1 h before TGF-β 1 treatment.

### Western blot

Frozen gastrocnemius and TA tissues were homogenized in RIPA buffer containing 1 mM PMSF, and Protease Inhibitor Cocktail (Roche, New Jersey). Lysates were centrifuged for 20 min at 12000×g (4 °C). Supernatants were transferred to a separate tube, and the bicinchoninic acid assay (BCA) was used for protein level quantification. Proteins were separated by SDS-PAGE gels (Beyotime, China), transferred to PVDF membranes (Millipore, USA), and blots were blocked for 1 h with 5% nonfat dry milk in TBS at room temperature. Primary antibodies against the following targets were proceeded overnight at 4 °C: mouse anti- myosin heavy chain (MHC; 1:3000; R&D Systems, USA), rabbit anti-LC3B (1:500; Abcam, UK), mouse anti-p62 (1:2000; Abcam, UK), rabbit anti-TGF-β1 (1:200; Abcam, UK) and rabbit anti-HMGB1 (1:10,000; Abcam, UK). After 3 washes, blots were incubated with appropriate secondary antibodies (Abcam, USA) at room temperature for 1 h. An ECL detection reagent and X-ray film were used for protein detection. Similar procedures were carried out for C2C12 myotubes.

### Wet weight and hematoxylin-eosin (HE) staining

Both the operational and contralateral sides of the gastrocnemius and TA were collected and saline was used to remove superficial blood. Filter paper was used to dry these samples, which were then weighed. The wet weight ratio was defined as the muscle weight of the operational side divided by the weight of the contralateral side. Then the samples were fixed with paraformaldehyde (4%), dehydrated, and paraffin embedded. Cross-sectional 4-μm thick slices of the muscle were prepared and stained with HE (Bioyear, China) to observe the pathological changes of atrophy.

### Immunofluorescence

Paraffin-embedded gastrocnemius and TA were cross-sectionally cut into 4-μm sections as above. After antigen retrieval, permeabilization, and goat serum blocking, primary antibody incubation was conducted overnight at 4 °C; primary antibodies included: mouse anti-MHC (1:1000), rabbit anti-TGF-β1 (1:100), rabbit anti-LC3B (1:200), rabbit anti-wheat germ agglutinin (WGA; 1:50; Abcam, USA) and rabbit anti-HMGB1 (1:400). Samples were then stained for 1 h with secondary Alexa Fluor 488- or Alexa Fluor 594-conjugated anti-mouse or anti-rabbit secondary antibodies (1:300, Invitrogen, USA), followed by a 5 min DAPI/PI (Sigma, USA) staining. Samples were then imaged using a fluorescence microscope. Similar procedures were carried out for C2C12 myotubes.

### Autophagy detection with ad-GFP-LC3B

C2C12 cells were placed into 6 well plates on sterile coverslips and allowed to differentiate for 7 days. Differentiation media was then replaced by new complete growth media, and 40 μL of Ad-GFP-LC3B was added per well. After a 24 h incubation, cells were used for subsequent experiments. Green puncta were detected using a fluorescence microscope, assessing at least 50 cells from each individual experiment after different treatments.

### Quantification of Myotube/fibre diameter

Myotube diameters were calculated based on immunofluorescent images of myotubes on multiple sections of a given coverslip, assessing at least 100 total myotubes with the ImageJ software package (National Institutes of Health, USA). The myotube diameter was determined at three points along the length of the myotube in a blinded fashion, and the average diameter per myotube was expressed as the mean of three measurements. Myotubes were defined as all multinucleated cells positive for the MHC stain and containing at least three nuclei. To measure fibre diameters, gastrocnemius muscle section images stained with WGA were analyzed, with fibres being selected manually, and the ImageJ software used to quantify the minimal Feret’s diameter for each.

### RNA sequencing (RNA-Seq)

Two weeks post-operation, three mice were sacrificed and samples (three normal gastrocnemius from contralateral side and three atrophy gastrocnemius from operational side) were immediately frozen using liquid nitrogen. Total RNA was isolated from samples using the Trizol (Invitrogen) according to the manufacturer’s protocol. Briefly, rRNAs were removed from Total RNA using EpicentreRibo-Zero rRNA Removal Kit (Illumina, USA) and fragmented to approximately 200 bp. Subsequently, the purified RNAs were subjected to first strand and second strand cDNA synthesis following by adaptor ligation and enrichment with a low-cycle according to instructions of NEBNext® Ultra™ RNA Library Prep Kit for Illumina (NEB, USA). The purified l library products were diluted to 10 pM for cluster generation in situ on the pair-end flow cell followed by sequencing (2 × 150 bp) HiSeq3000. HTSeq was subsequently employed to convert aligned short reads into read counts for each gene model. Differential expression was assessed by DEseq using read counts as input. The Benjamini-Hochberg multiple test correction method was enabled. Differentially expressed genes were chosen according to the criteria of fold change > 2 and adjusted *p*-value < 0.05.

### Enzyme-linked immunosorbent assay

To determine HMGB1 release from cultured cells, supernatants were collected after different treatment and centrifuged to remove cellular debris. The samples were analyzed using a mice HMBG1 enzyme-linked immunosorbent assay (ELISA) kit (CUSABIO, China) following the manufacturer’s instructions. Sample absorbance was measured in triplicate within 5 min after adding the Stop Solution using a plate reader at 450 nm.

### Small interfering RNA (SiRNA) silencing

C2C12 cells were seeded at 2 × 105 cells/mL in 6-well plates and the siRNA-HMGB1 transfection was performed when cell confluency reached 50%. The double-stranded siRNA for the HMGB1 target sequence was: sense 5′-CAAGGCUCGUUAUGAAAGATT -3′ and antisense 3′-TTGUUCCGAGCAAUACUUUCU -5′. An siRNA non-specific control was also used. Transfection were operated according to standard transfection protocols for cell cultures using Lipofectamine 2000 reagent (Invitrogen). The expression of HMGB1 was subsequently evaluated using immunofluorescence and western blot. For preparation of HMGB1 knock-down study in gastrocnemius in the operation side, cholesterol-conjugated HMGB1 siRNA (10 nmol) and its negative control in 0.1 mL saline buffer was injected into gastrocnemius once every 3 days for 2 weeks after denervation as reported previously [36].

### Statistical analysis

The GraphPad Prism 5 software (GraphPad Software, USA) was used for all analyses. Results are shown as mean ± standard deviation for at least three independent experiments. ANOVA with a post-hoc Dunnett’s test was used for all comparisons. *P* < 0.05 was the threshold of statistical significance.

## Results

### The dynamic changes of TGF-β1 expression and autophagic activity during denervation-induced atrophy

**Fig. 1:**
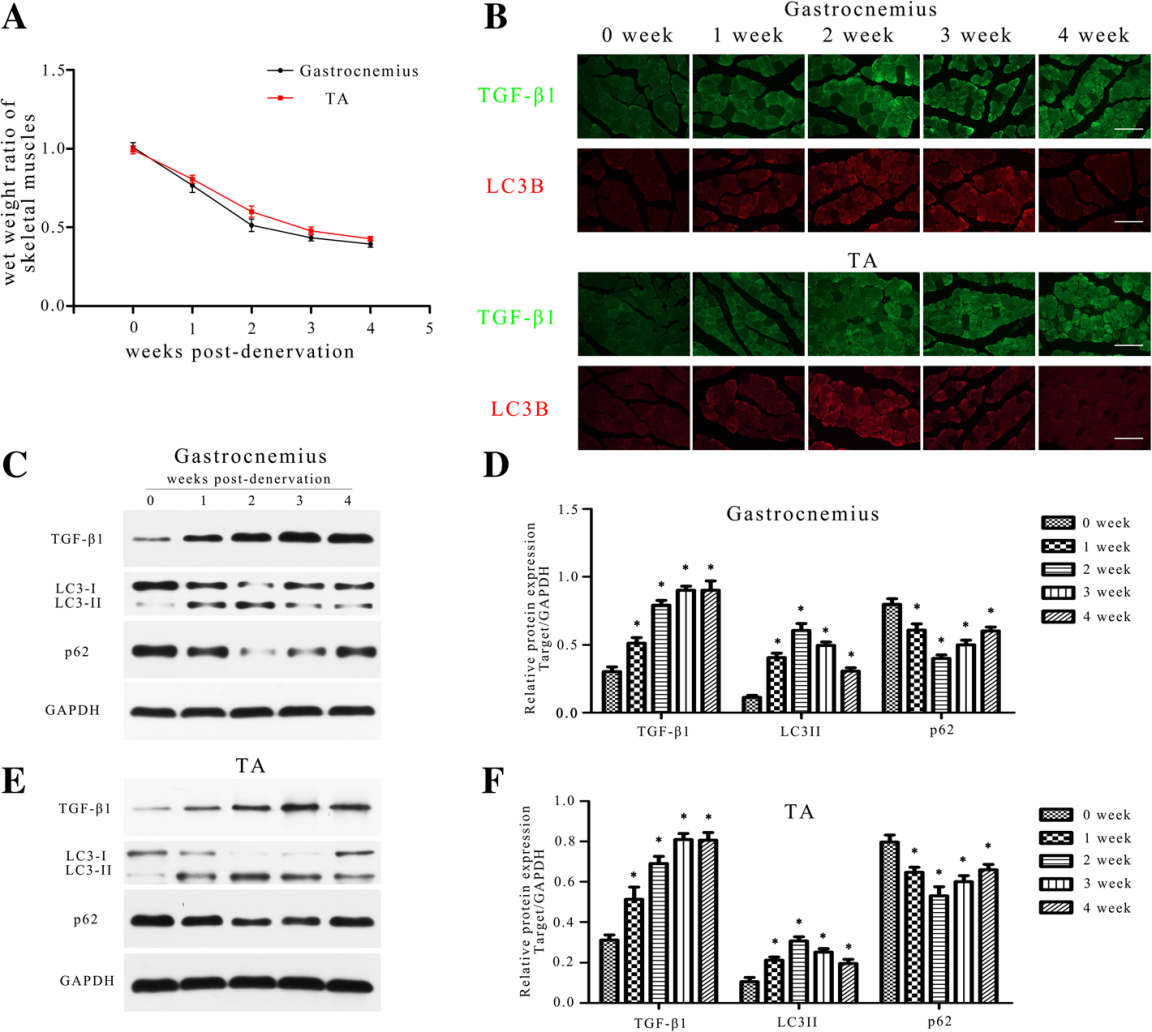
Dynamic changes of TGF-β1 expression and autophagic activity during denervation-induced atrophy. **a** The wet weight ratio (the weight of the operational side divided by the contralateral side) of gastrocnemius and TA at indicated time points post-denervation. **b** Immunofluorescence staining of TGF-β1 and LC3B in gastrocnemius and TA harvested at indicated time points post-denervation. *Scale bar* 50 μm. **c**, **d** Western blot analysis of TGF-β1, LC3B and p62 protein expression in gastrocnemius at different time after denervation. Relative grey values analyses were performed. **e**, **f** Western blot analysis of TGF-β1, LC3B and p62 protein expression in TA at different time after denervation. Relative grey values analyses were performed. Every 5 mice were sacrificed at each time point, *P < 0.05 vs control (0 week)

We initially sought to investigate whether the expression of TGF-β1 and autophagic activity are altered in denervation-induced atrophy. Consistent with previous research [16, 37], TA and gastrocnemius of the operation side rapidly atrophied after denervation surgery. Weight measurements of the muscle revealed that the atrophic process was biphasic, with a rapid loss (average 49% in gastrocnemius) in muscle mass over the first 2 weeks and then a more gradual reduction (average 12% in gastrocnemius) over the following 2 weeks (Fig. 1a). Immunofluorescence staining and western blots results showed that the TGF-β1 expression increased substantially during the first 2 weeks post-denervation, with slight changes during the second phase (Fig. 1b-f). The LC3-II (a quantitative index of autophagy) increased in a manner consistent with TGF-β1 during the first 2 weeks, and then decreased gradually during the following 2 weeks, suggesting that the underlying autophagic process is also biphasic (Fig. 1b-f). As expected, the p62 changed to the contrary of LC3-II (Fig. 1c-f). These results confirmed that TGF-β1 and autophagy are linked to the process of denervation-induced muscle atrophy. As the period (first 2 weeks) of rapid loss of muscle mass coincided with an increase in TGF-β1 expression and autophagic activity, we next wanted to explore the role of TGF-β1 and autophagy in denervation-induced atrophy. Gastrocnemius, with a more significant increase of LC3-II than TA, were targeted for the next experiments.

### The atrophic effect of TGF-β1 in denervated muscle

**Fig. 2:**
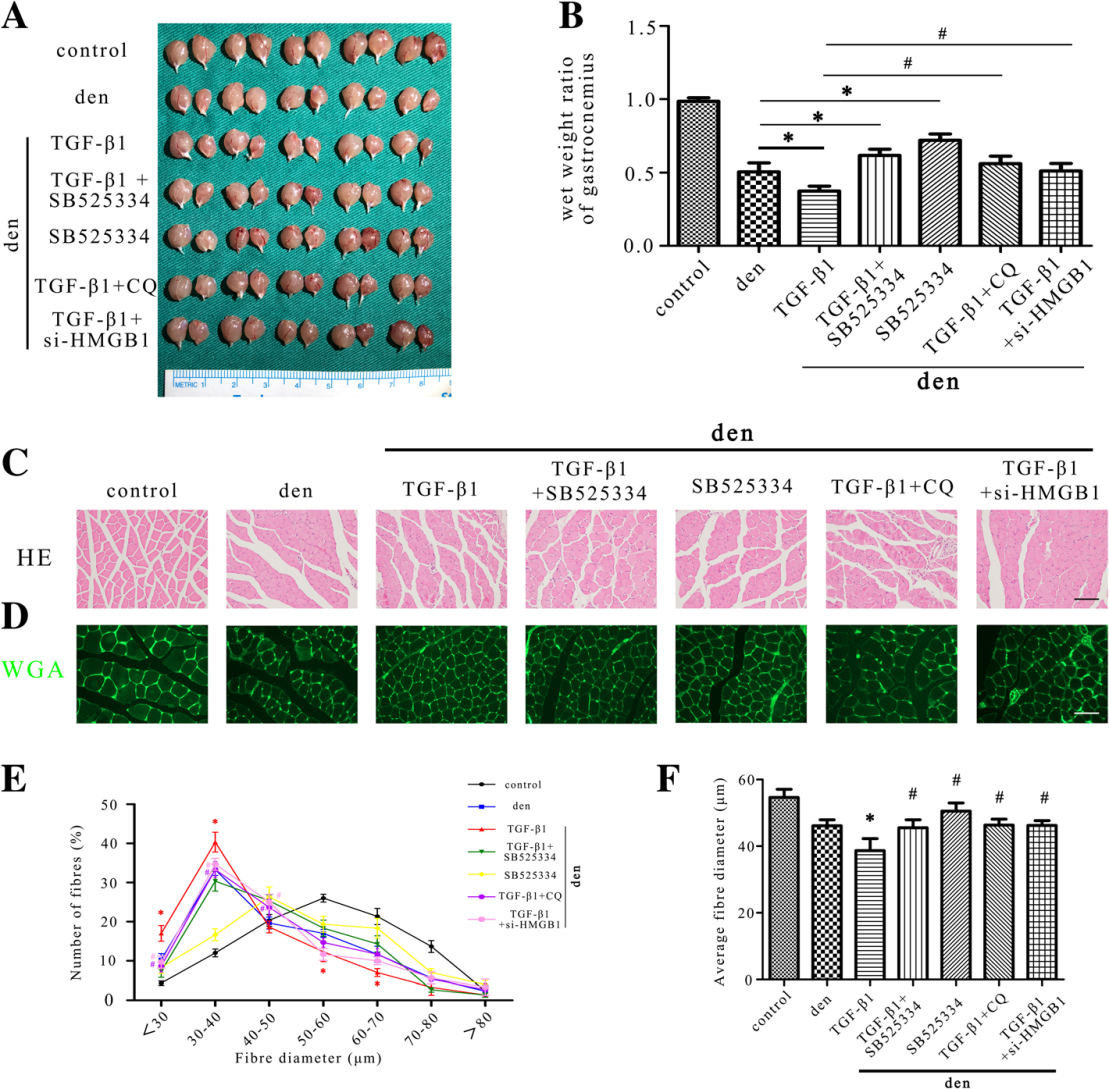
Atrophy promoting effect of TGF-β1 in gastrocnemius. **a** Gastrocnemius of different groups harvested 2 weeks post-denervation. **b** The wet weight ratio of gastrocnemius in different groups. **c** Morphological observation of gastrocnemius muscles in different groups by HE staining. *Scale bar* 50 μm. **d**-**f** Quantification of muscle fibres diameter by immunofluorescence staining of WGA. 5 mice/group, *Scale bar* 50 μm. *P < 0.05 vs denervation only group. ^#^*P* < 0.05 vs denervation + TGF-β1 group. Den, denervation

To delineate the role of TGF-β1 in skeletal muscle following long-term denervation, the sciatic nerve of the mice was cut and gastrocnemius were analyzed 2 weeks post operation (Fig. 2a). Muscle mass evaluation and HE staining showed that the administration of TGF-β1 could significantly aggravated muscle atrophy while this effect was attenuated by the inhibitor of TGF-β (SB525334) (Fig. 2b and c) (Considering the possible compensatory hypertrophy, the wet weights of gastrocnemius muscles of the non-operation limbs had also been statistically analysed, no differences were found in different groups). We further analyzed fibre diameter and observed that diminution in muscle fibre size was induced by TGF-β1 and could be partially prevented when SB525334 was administered (Fig. 2d-f). The TGF-β1 injections produced a shift towards fibre of a lesser diameter than the control group, while in the presence of SB525334 this effect was lost.

**Fig. 3:**
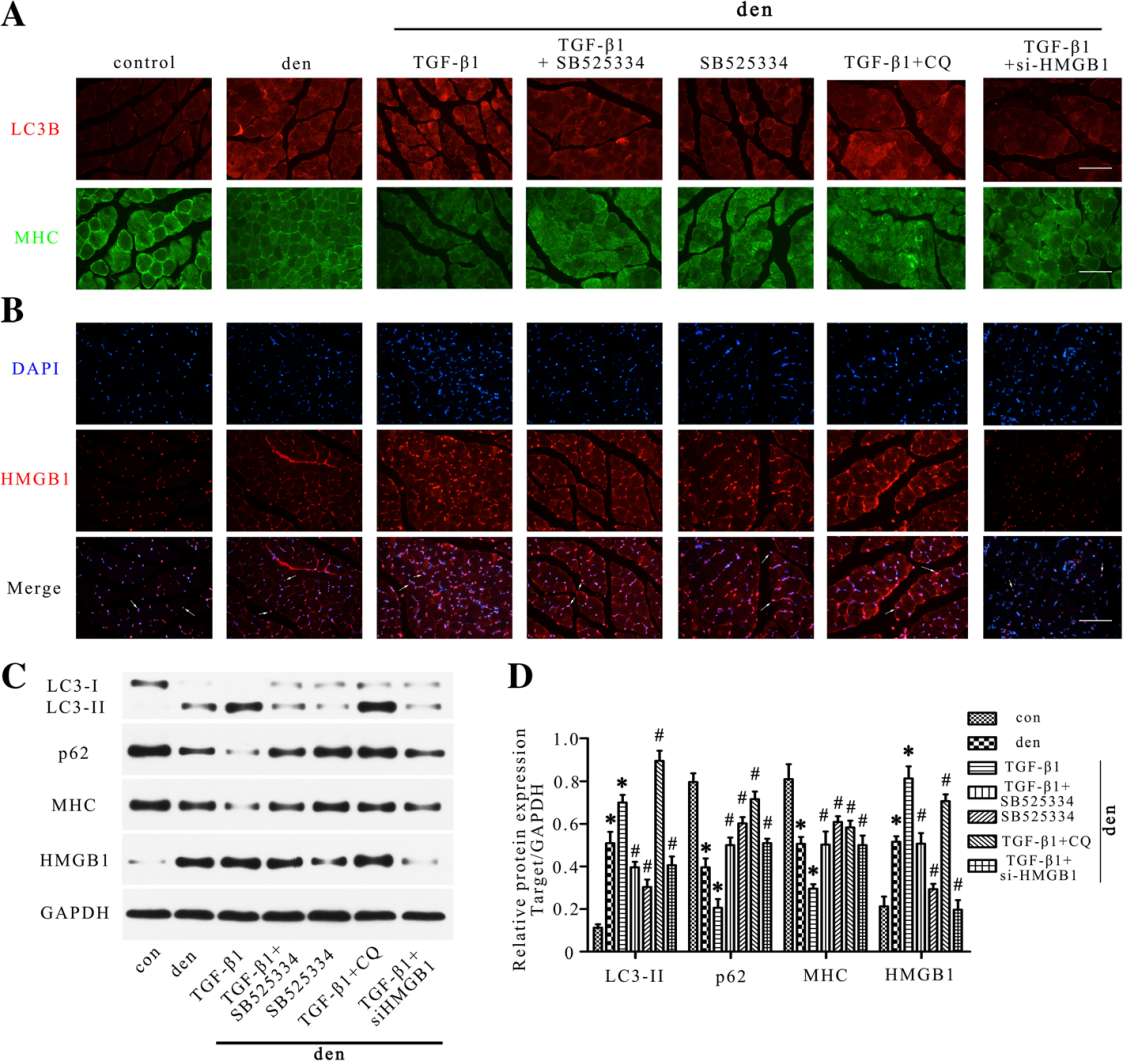
HMGB1/autophagy pathway mediated the atrophic effect of TGF-β1 in denervated skeletal muscle. **a** Immunofluorescence staining of LC3B and MHC in gastrocnemius in different groups. *Scale bar* 50 μm. **b** HMGB1 staining of gastrocnemius. The arrows indicated HMGB1-positive staining. *Scale bar* 50 μm. **c** Western blot analysis of LC3B, p62, HMGB1 and MHC protein expression in gastrocnemius muscles. **d** Relative grey values analyses of the western blot results. 5 mice/group, *P < 0.05 vs control, ^#^*P* < 0.05 vs denervation + TGF-β1 group. Den, denervation; Con, control; DAPI, 4′,6-diamidino-2-phenylindole

The levels of myofibrillar proteins, such as MHC, were assessed in different conditions to by immunofluorescence staining and western blot (Fig. 3a, c and d). Showing an opposite manner to TGF-β1, the MHC levels decreased with the atrophy of gastrocnemius and was further depressed when TGF-β1 was administrated, while this effect was attenuated by inhibitor of TGF-β1. Totally, these results indicate that TGF-β1 was capable of promoting denervation-induced atrophy.

### Autophagy was a pivotal downstream mechanism in TGF-β1-induced atrophy

To further characterize the mechanism of the atrophy promoting effect of TGF-β1, immunofluorescence staining and western blot were performed and the expression of LC3B, p62 and MHC were quantified (Fig. 3a, c and d). Significant activation of autophagy was monitored following 2 weeks of denervation and systemic administration of TGF-β1 could further enhance the autophagic activity, while SB525334 almost abolished the further activation of autophagy induced by TGF-β1. Besides, in wet weight, HE staining and fibre diameter analysis (Fig. 2), the atrophic effect of TGF-β1 was reversed when autophagy was blocked by CQ. The MHC level was also up-regulated when TGF-β1 was treated together with CQ compared to TGF-β1 only (Fig. 3a, c and d). Taken together, these evidences suggested that autophagy is a pivotal downstream mechanism in TGF-β1-induced atrophy.

### RNA-Seq in atrophied gastrocnemius

**Fig. 4:**
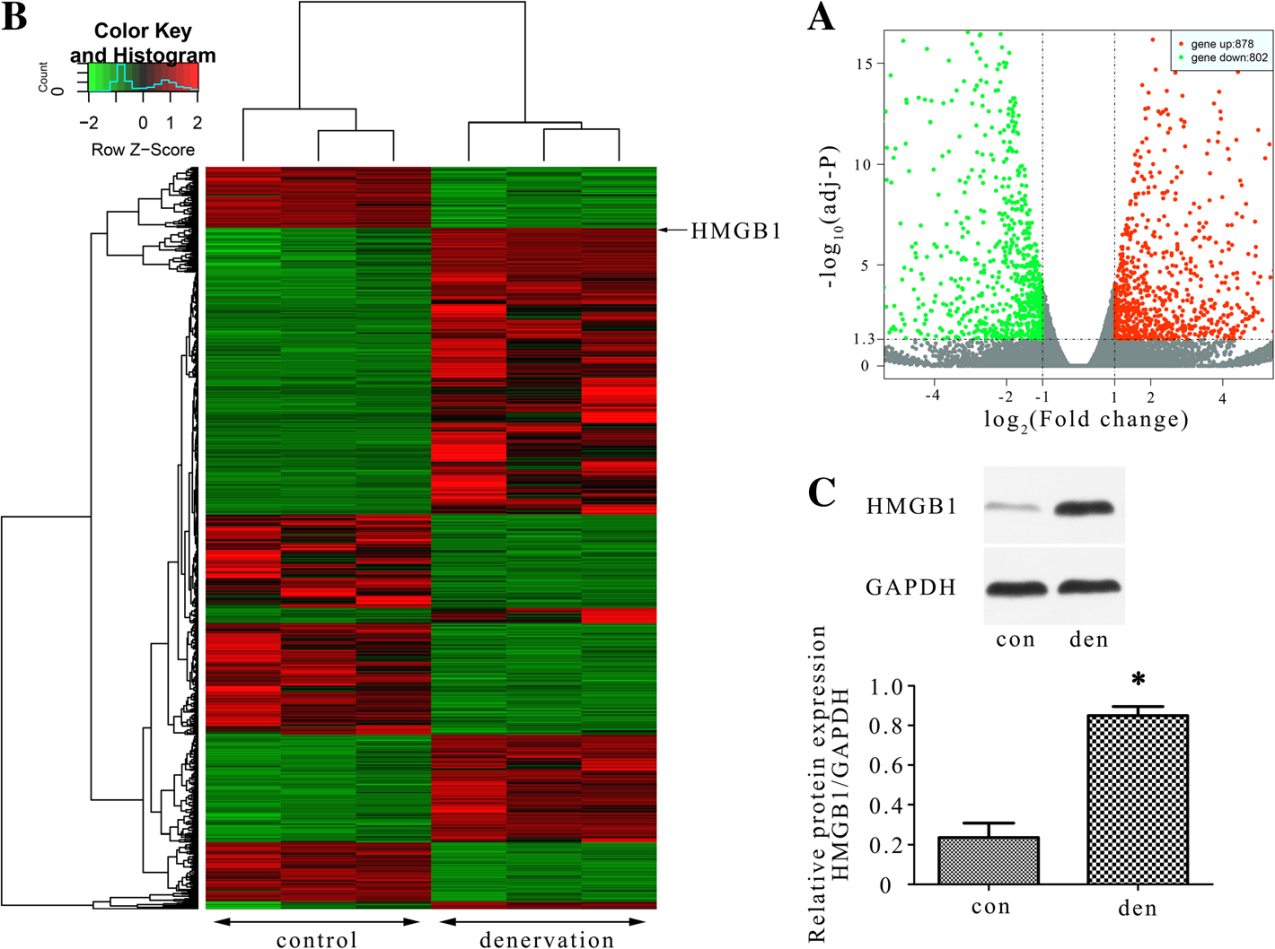
Differential expression of genes in gastrocnemius. **a** Volcano plots were constructed using fold change values and adjusted *p*-values. The vertical lines corresponded to 2.0-fold up- and downregulation between the normal and atrophy groups, and the horizontal line represented the adjusted *P*-value = 0.05. The red and green point in the plot represented the differentially expressed genes showing statistical significance. The arrows indicated HMGB1. **b** Hierarchical cluster analysis of the significantly upregulated and downregulated genes (|log_2_(fold_change)| > 2 and adjusted *p*-value < 0.05). Each column represented a sample and each row represented a gene. The expression levels were presented in different colors indicating expression levels above and below the median expression level across all samples. **c** Western blot analysis was performed to verify the RNA-Seq results of HMGB1 in gastrocnemius of contralateral side and denervation side. *P < 0.05 vs contralateral side. Den, denervation; Con, control

To further explore the possible proteins or signal pathways that works during skeletal muscle atrophy, RNA-Seq was carried out and the volcano plots showed the variation of gene expression between atrophied gastrocnemius and control (Fig. 4a). In total, 1680 differentially expressed genes were identified, in which 878 genes were upregulated and 802 genes were downregulated. Hierarchical cluster analysis showed the differentially expressed genes over 4-fold change (Fig. 4b). HMGB1 was shown in the cluster heat map and was upregulated by nearly 4.7-fold. Considering its specific autophagy regulating effect reported by recent studies [31, 38], we paid great attention to this well-known damage associated molecular patterns (DAMPs): HMGB1. To verify the RNA-Seq results, western blot analysis (Fig. 4c) of HMGB1 were performed. In accordance with RNA-Seq results, the expression of HMGB1 was up-regulated in atrophied gastrocnemius compared with control (no denervation). Above results indicated that HMGB1 was up-regulated in atrophied gastrocnemius and may be interrelated with the occurrence of denervation-induced atrophy.

### HMGB1/autophagy pathway mediated the atrophic effect of TGF-β1 in denervated skeletal muscle

HMGB1 had been reported to regulate autophagy activity in various models [39, 40]. Here we investigated the interaction between TGF-β1, HMGB1 and autophagy in denervation-induced muscle atrophy by immunofluorescence staining and western blot. As seen in Fig. 3, HMGB1 was up-regulated and released from nucleus to cytoplasm and extracellular space in the denervation group and was further elevated by TGF-β1 treatment, while SB525334 blocked the further increase of HMGB1 induced by TGF-β1. Besides, treatment with TGF-β1 combined with si-HMGB1 obviously enhanced the expression of MHC (Fig. 3) and improved muscle atrophy compared with TGF-β1 only group (Fig. 2). Above results demonstrated that HMGB1 was involved in the atrophic effect of TGF-β1.

To find out whether autophagy was modulated by HMGB1 in atrophic muscle, we evaluated the effect of si-HMGB1 on the autophagy activity. Autophagy was activated in atrophic muscle and was further enhanced by TGF-β1, while the addition of si-HMGB1 reversed the activation of autophagy, which was seen in both immunofluorescence staining and western blot experiments (Fig. 3). Taken together, these findings indicated that HMGB1/autophagy pathway illuminated the atrophic effect of TGF-β1 in denervated skeletal muscle.

### Atrophic effect of TGF-β1 in vitro

**Fig. 5:**
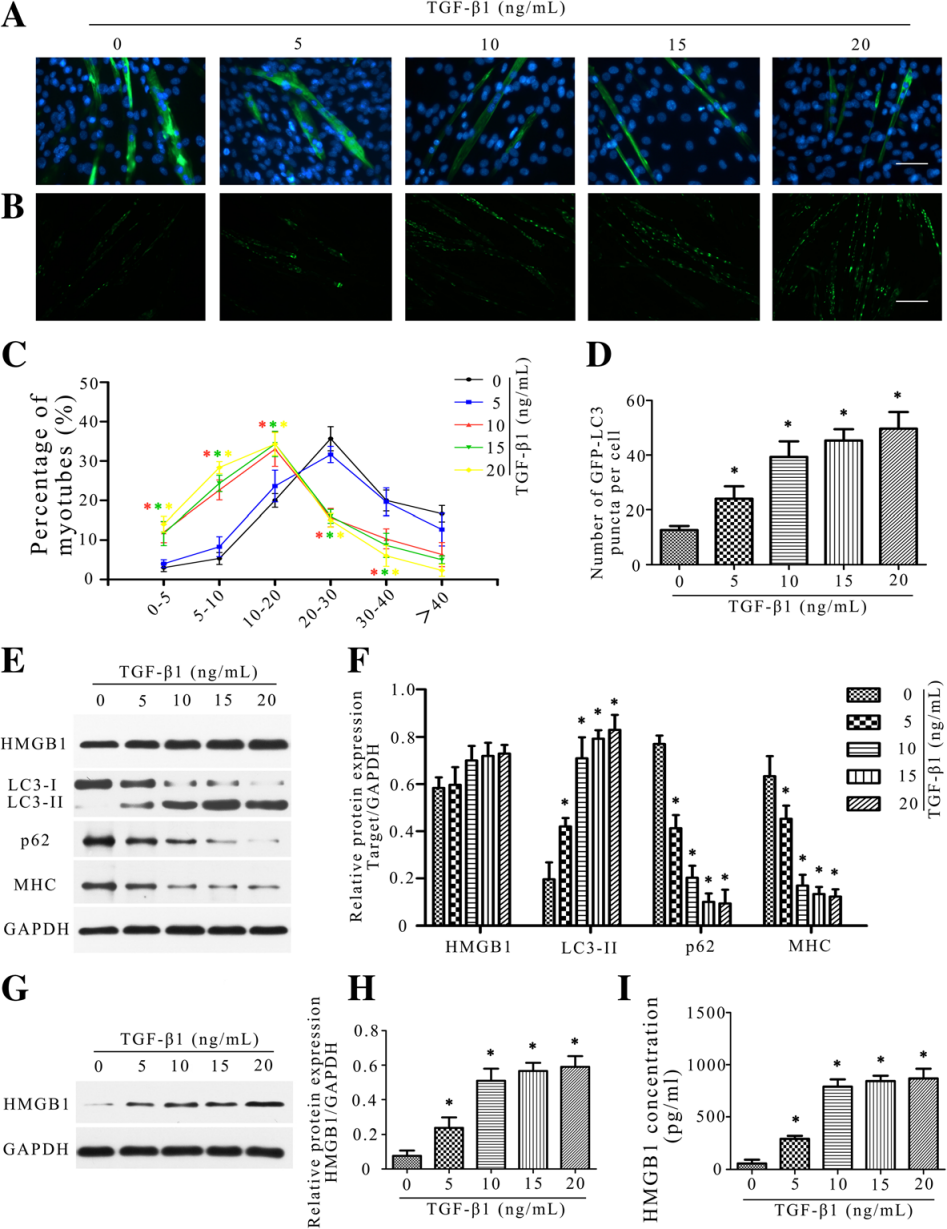
Atrophic effect of TGF-β1 in vitro. **a**, **b** Quantification of C2C12 myotube diameter by immunofluorescence staining of MHC after treated with a dose gradient of TGF-β1. One-hundred total myotubes were evaluated in each group with the ImageJ software. *Scale bar* 50 μm. **c** Representative fluorescent images of C2C12 myotubes expressing GFP-LC3 after TGF-β1 treatment. *Scale bar* 100 μm. **d** Quantification of the average number of GFP-LC3 puncta per cell as described in (**c**). **e**, **f** Western blot analysis of HMGB1, LC3B, p62 and MHC protein expression in C2C12 myotubes. Relative grey values analyses were performed. **g**, **h** Western blot analysis of HMGB1 in the supernatant after TGF-β1 treatment. **i** ELISA analysis of HMGB1 in the supernatant after TGF-β1 treatment. The values were obtained from three independent experiments. *P < 0.05 vs control (0 ng/ml)

To further confirm the atrophic effect of TGF-β1 on skeletal muscle in vitro, a dose gradient of TGF-β1 was added to C2C12 myotubes. As shown in Fig. 5a and b, TGF-β1 treatment increased the proportion of myotubes with small diameters ([5–10 μm] and [10–20 μm]) and decreased the proportion with big diameters ([20–30 μm] and [30–40 μm]) in a dose-dependent manner compared to the control (0 ng/ml). Activation of autophagy was also observed after TGF-β1 treatment (Fig. 5c-f). Western blot and ELISA analysis showed similar results (Fig. 5e-i), the expression of HMGB1 and LC3-II increased, p62 and MHC decreased following TGF-β1 treatment both in a dose-dependent manner (HMGB1 was significantly higher in the supernatant of TGF-β1-treated cells shown by both ELISA and western blot (Fig. 5g-i), despite that no obvious change was found in cell lysates). The effect of TGF-β1 on C2C12 myoblast was also explored, similar trends of HMGB1, LC3-II and p62 were found, despite no obvious change in shape and size (Additional file 1: Figure S1). 10 ng/ml TGF-β1 was then used for the following experiments considering the obvious atrophic effect achieved.

### TGF-β1 induced C2C12 myotube atrophy through HMGB1/autophagy pathway

**Fig. 6:**
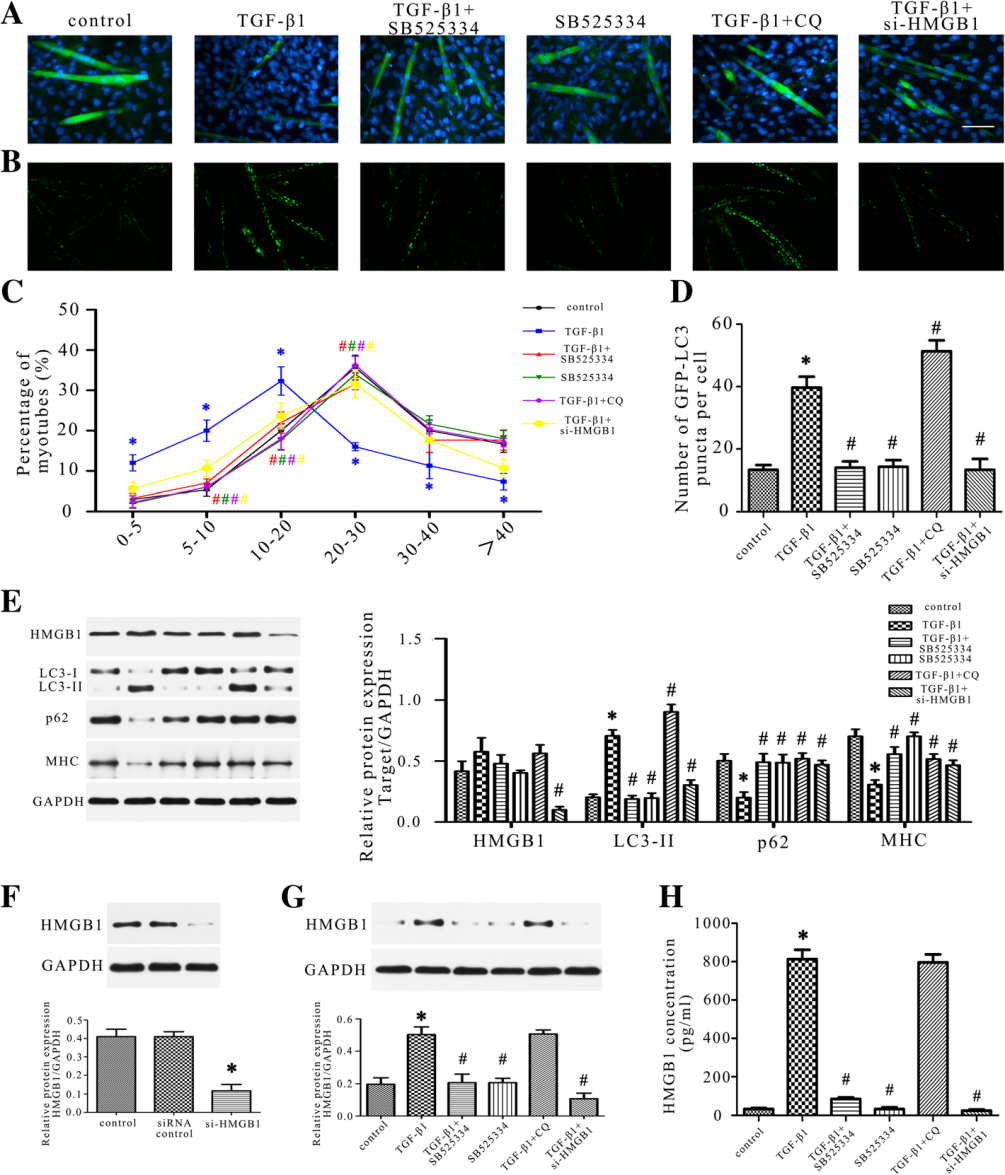
TGF-β1 induced C2C12 myotube atrophy through HMGB1/autophagy pathway. **a**, **b** Quantification of C2C12 myotube diameter by immunofluorescence staining of MHC. Scale bar 50 μm. **c** Representative fluorescent images of C2C12 myotubes expressing GFP-LC3 in each group. Scale bar 100 μm. **d** Quantification of the average number of GFP-LC3 puncta per cell as described in (**c**). **e** Western blot analysis of LC3B, p62, MHC and HMGB1 protein expression in C2C12 myotubes. Relative grey values analyses were performed. **f** The effect of si-HMGB1 in C2C12 cells was evaluated by western blot. **g** Western blot analysis of HMGB1 in the supernatant of different groups. **h** ELISA analysis of HMGB1 in the supernatant of different groups. The values were obtained from three independent experiments. *P < 0.05 vs control, ^#^*P* < 0.05 vs TGF-β1 group

To explore the involved mechanism of the atrophic effect of TGF-β1, C2C12 myotubes were studied under different treatment. Similar to in vivo studies, TGF-β1 raised the HMGB1 expression, activated autophagy and facilitated myotube atrophy while SB525334 reversed these changes, which was shown by western blot, ELISA, LC3 puncta and myotube diameter analysis (Fig. 6). CQ inhibited autophagy and eliminated the atrophic effect of TGF-β1 also. Si-HMGB1 treatment blocked the activation of autophagy induced by TGF-β1 and subsequently improved the myotube atrophy (Fig. 6). All of these results indicated that TGF-β1 induced C2C12 myotube atrophy through HMGB1/autophagy pathway.

## Discussion

Peripheral nerve injuries are a growing topic of interest, particularly in developing countries where workers often suffer disabilities stemming from peripheral nerve injury complications such as limb weakness or muscle atrophy [41–43]. Unfortunately, no clinical treatment for muscle atrophy has yet been discovered, making it vital to clarify the exact mechanisms by which denervation-induced skeletal muscle atrophy progresses in order to identify promising therapies.

Hmg family was discovered as a group of non-histone nuclear proteins with high electrophoretic mobility and contained two DNA-binding HMG-box domains and an acidic C-terminal tail [44]. HMGB1, which was the most abundant and well-studied member among Hmg family, might exert an important function for chromatin structure and further influence the transcription, replication, recombination, DNA repair and genomic stability in the nucleus [44, 45]. In addition to its nuclear role, HMGB1 could also act as an extracellular signaling molecule to modulate the inflammation, immune response, autophagy and cancer [31, 45, 46]. Besides, recent researches have demonstrated that HMGB1/autophagy pathway is involved in several disease models, such as liver fibrosis, Parkinson’s disease and so on [39, 40]. In our research, we explored the role of HMGB1 in denervation-induced muscle atrophy. The data showed translocation and up-regulation of HMGB1 in atrophic muscles and C2C12 myotubes. The regulation effect of HMGB1 on autophagy was also confirmed by si-HMGB1 treatment.

The ability of TGF-β1 to drive skeletal muscle atrophy is well documented [27, 30]. TGF-β1 levels have also been shown to be increased in the context of some skeletal muscle conditions, including Duchenne muscular dystrophy [29, 47]. TGF-β1 signaling is also elevated in models of Ang-II- or disuse-induced muscle atrophy [48–50]. Blocking TGF-β in a cancer cachexia model has also supported a function for TGF-β in atrophic disease conditions [51]. We similarly found that TGF-β1 was up-regulated in denervation-induced skeletal muscle atrophy, and exogenous injection of TGF-β1 worsened this atrophy. The ability of TGF-β1 to drive atrophy was further explored in vitro using C2C12 myotubes, yielding similar results. TGF-β downstream signaling pathways have been well characterized, such as those dependent on Smad 2/3 phosphorylation that allow for gene regulation via formation of a multimeric complex with Smad 4 [52]. Other signaling pathways including ERK1/2 and JNK1/2 have also been found to be activated by TGF-β1 in skeletal muscle atrophy [53]. UPS and autophagy are the two major mechanisms mediating protein degradation in skeletal muscle cells, and both are regulated by a range of upstream pathways [54]. Recent evidence has indicated that TGF-β can induce overactivation of the UPS by increasing E3 ubiquitin ligase MuRF-1 expression in muscle atrophy [55, 56]. Less well characterized is the relationship between TGF-β and autophagy.

Despite being counter-intuitive, since autophagy may appear to be an catabolic pathway, autophagy is vital for maintaining protein homeostasis in a wide range of cell types [57–59]. While studying autophagy is not an easy task due to its very dynamic nature, some recent researches revealed its dual effect in different situations. Under pathological conditions, autophagy increases are associated with muscle wasting induced by pro-atrophic stimuli, fasting, high-fat diet/insulin resistance, hypoxia, and exercise [60]. Besides, suppression of autophagy has also been proved to be deleterious in some cases. Indeed, autophagic flux was recently found to be blocked (characterized by high LC3 II and high p62) in cachexia -associated muscle atrophy and restoring of the autophagic flux (characterized by high LC3 II and low p62) improved muscle mass and function [61]. Similarly, Sod1^−/−^ mice (characterized by reduced Sarcoplasmic Reticulum Ca2+ ATPase activity, muscle weakness and atrophy) maintained a suppressed baseline autophagic flux but reversed muscle atrophy through enhancement of autophagy by CDN1163 treatment [62]. Analogous situation was also found in tumor development, diabetes, or chronic inflammation [63–65], confirming the dual character of autophagy. In the present study, we determined that autophagy was activated in the context of denervation-induced skeletal muscle atrophy, and that it was further activated by TGF-β1, leading to more weight loss and smaller fibre diameter, which could be reversed when autophagy was blocked by CQ. Autophagy was similarly activated in C2C12 myotubes that were subjected to TGF-β1 treatment, leading to smaller myotube diameter and decreased MHC expression. When the TGF-β1 receptor was blocked using a specific inhibitor, activation of autophagy was also attenuated both in vivo and in vitro, with an accompanying reversal of the atrophic effects of TGF-β1 in skeletal muscle. Furthermore, TGF-β1/HMGB1/autophagy modulating axis in muscle atrophy was confirmed in our research. Exogenous TGF-β1 and its inhibitor (SB525334) were administrated in vivo and in vitro, HMGB1 was consequently up-regulated, accompanied by activation of autophagy, leading to progressive muscle atrophy.

In our preliminary experiment (Fig. 1), autophagy in gastrocnemius seemed more sensitive than TA after denervation (showing higer LC3-II), this result is consistent with the findings of previous researches, showing different mechanisms for fiber-type specificity of skeletal muscle atrophy [66, 67]. The preliminary experiment also showed the time-dependent activation of autophagy (activated in the first 2 weeks and attenuated in the next 2 weeks) after denervation, this is really interesting as blocking of autophagy has been proved to be helpful for muscle atrophy in the first 2 weeks while ineffective after 4 weeks (showing by Pigna [68]), from which we speculate that autophagy works mainly in the early stage (first 2 weeks) after denervation and it is not the only causation of gastrocnemius atrophy, further study may be necessary for our group to validate this conjecture.

Given the complex microenvironment in which the skeletal muscle atrophy occurs following denervation, no suitable in vitro model has been established to imitate this denervated state. Hence the C2C12 myotubes were used for all in vitro studies.

## Conclusions

To summarize, this study demonstrates that TGF-β1 could promote atrophy in denervated muscle, and HMGB1/autophagy pathway mediated the atrophic effect of TGF-β1. The present findings contributed previously unknown insights into the mechanisms underlying the role of TGF-β1 in muscle atrophy. The findings also advanced the HMGB1/autophagy pathway as a potential target to improve muscle atrophy in patients with peripheral nerve injury.

## Additional file

Additional file 1: **Figure S1.** Effect of TGF-β1 on C2C12 myoblast. (A) Photos showing the appearance of C2C12 myoblast in TGF-β1(−) and TGF-β1(+) groups, cells were exposed to 10 ng/ml TGF-β1 for 72 h. (B) Western Blot analysis of cell lysates. (C) Western Blot analysis of the supernatant. (D) ELISA analysis of HMGB1 in the supernatant of different groups. The values were obtained from three independent experiments. *P < 0.05 vs TGF-β1(−) group. (JPG 784 kb)

## Abbreviations

BCA: Bicinchoninic acid assay
CQ: Chloroquine
DMEM: Dulbecco’s modified Eagle’s medium
ECM: Extracellular matrix
ELISA: Enzyme-linked immunosorbent assay
FBS: Fetal bovine serum
HE: Hematoxylin-eosin
HMGB1: High-mobility group box-1
MHC: Myosin heavy chain
RNA-Seq: RNA Sequencing
SiRNA: Small interfering RNA
TA: Tibialis anterior
TGF-β1: Transforming growth factor beta 1
UPS: Ubiquitin-proteasome system
WGA: Wheat germ agglutinin
