# Supplementary figures and images for: HMGB1/autophagy pathway mediates the atrophic effect of TGF-β1 in denervated skeletal muscle
Source: Cell Commun Signal. 2018 Dec 7;16:97. doi: 10.1186/s12964-018-0310-6 (PMC6286536; doi:10.1186/s12964-018-0310-6)

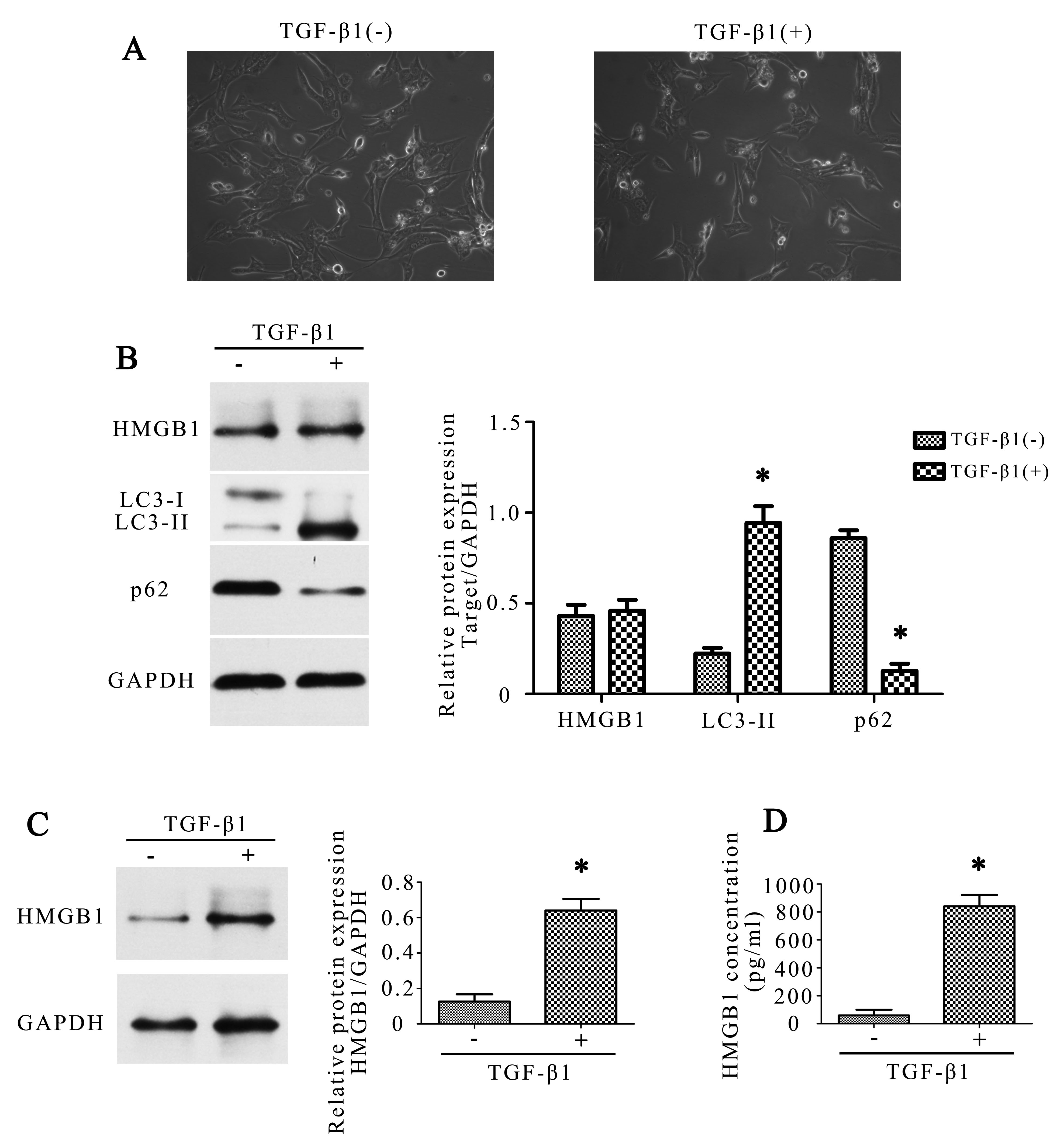

Supplement: Supplementary file 1 — Figure S1. Effect of TGF-β1 on C2C12 myoblast. (A) Photos showing the appearance of C2C12 myoblast in TGF-β1(−) and TGF-β1(+) groups, cells were exposed to 10 ng/ml TGF-β1 for 72 h. (B) Western Blot analysis of cell lysates. (C) Western Blot analysis of the supernatant. (D) ELISA analysis of HMGB1 in the supernatant of different groups. The values were obtained from three independent experiments. *P < 0.05 vs TGF-β1(−) group. (JPG 784 kb) [file 12964_2018_310_MOESM1_ESM.jpg]
